# South African Speech-Language Therapy and Audiology students’ experiences of academic and clinical curriculum transformation

**DOI:** 10.4102/sajcd.v72i1.1086

**Published:** 2025-05-27

**Authors:** Farieda Abrahams, Katijah Khoza-Shangase

**Affiliations:** 1 Department of Audiology, School of Human and Community Development, University of the Witwatersrand, Braamfontein, South Africa

**Keywords:** speech-language therapy, audiology, curriculum transformation, cultural competence, clinical training, South Africa, Afrocentric approaches, indigenous languages

## Abstract

**Background:**

South African Speech-Language Therapy and Audiology (SLH) programmes historically followed Western frameworks, often lacking in cultural and linguistic relevance to the local context. SLH curricula and clinical training continue to face challenges in aligning with South Africa’s diverse population and healthcare needs.

**Objectives:**

To investigate South African undergraduate SLH students’ experiences of transformation in the curriculum and clinical service provision.

**Method:**

A cross-sectional survey design was employed on students from four South African universities. Data were collected through an online questionnaire. Quantitative data were analysed using descriptive and inferential statistics, while thematic analysis was conducted on qualitative responses.

**Results:**

Findings revealed that most students perceived limited cultural and linguistic relevance in the SLH curriculum, with 60% indicating insufficient Afrocentric content and South African language integration. Students reported feeling underprepared for working with diverse linguistic groups, citing a lack of appropriate resources and limited training in cultural competence. Students proficient in an indigenous language reported higher confidence levels in clinical interactions. Qualitative themes included lack of representation in curriculum content, insufficient cultural competence training and desire for indigenous knowledge and Afrocentric approaches.

**Conclusion:**

The study highlights critical gaps in SLH education in South Africa, particularly in the inclusion of culturally and linguistically relevant training. Recommendations include integrating Afrocentric content, indigenous language modules and practical training for working in diverse clinical settings.

**Contribution:**

This study contributes to the discourse on decolonising SLH education in South Africa, offering evidence-based recommendations to align training with the country’s diverse sociocultural and linguistic realities.

## Introduction

South Africa’s journey towards transformation in higher education and healthcare services is ongoing, complex and critical, especially in fields such as Speech-Language Therapy and Audiology (SLH). The necessity for a curriculum that reflects the country’s sociocultural and linguistic diversity is particularly pronounced, as SLH services require practitioners to effectively engage with clients across various linguistic and cultural backgrounds. Historically, SLH in South Africa has predominantly catered to a white minority, following Eurocentric frameworks and methodologies (Abrahams et al., 2023; Kathard & Pillay, 2013; Khoza-Shangase & Mophosho, 2018; Moonsamy et al., 2017). This emphasis has perpetuated the exclusion of indigenous knowledge, languages and contextual nuances, which are essential for equitable healthcare delivery and effective communication. The historical development of SLH professions under apartheid led to a predominance of Eurocentric, English- and Afrikaans-based curricula, which have long marginalised indigenous African languages and perspectives (Kathard & Pillay, 2013; Khoza-Shangase & Mophosho, 2018). Even decades into democracy, the SLH profession struggles to produce practitioners reflective of South Africa’s diverse population, which remains a majority of black Africans in demographic composition, with approximately 60% of the practitioners being classified as white (Pillay et al., 2020). Transformation efforts in South African higher education, particularly in fields like SLH, are critical not only to address this racial and cultural disconnect but also to ensure that the curriculum and clinical service models align with the linguistic, cultural and social realities of the country’s population.

The notion of transformation in South African higher education, and specifically within SLH, has been significantly influenced by post-apartheid reforms and movements like #FeesMustFall (Abrahams et al., 2023; Khunou et al., 2019; McIntyre-Mills, 2019). These have highlighted the lingering presence of institutional cultures that continue to marginalise black students and underrepresent African languages and culturally relevant materials (Khoza-Shangase & Kalenga, 2024a). As a result, a curriculum that does not sufficiently incorporate local languages and cultural perspectives has been identified as a barrier, leaving SLH students underprepared to work with the country’s diverse population (Abrahams et al., 2023). For instance, current SLH curricula remain heavily influenced by English and Afrikaans, with limited integration of African languages and indigenous knowledge (Khoza-Shangase et al., 2021; Pillay & Kathard, 2015). This disconnect risks reinforcing a form of ‘othering’ in the therapeutic process, as clients from linguistically and culturally diverse backgrounds may feel that therapy is not designed for them, and may then struggle to find culturally resonant support from clinicians who are not equipped with the linguistic or cultural tools to bridge these gaps. Without clinicians who can communicate in their home languages or incorporate culturally relevant therapy strategies, some clients may struggle to relate to the therapeutic process, potentially leading to disengagement or reduced treatment efficacy. In addition to linguistic and cultural relevance, curriculum transformation in South African SLH programmes must also consider the rural–urban divide in healthcare access and resource allocation. Many training institutions and clinical placements are situated in urban areas, where students have access to well-equipped facilities, diverse clinical resources and structured supervision. In contrast, rural healthcare settings often lack adequate SLH services, diagnostic tools and trained professionals, which impacts the quality and effectiveness of therapy. Without sufficient exposure to rural practice contexts, students may graduate unprepared to navigate the challenges of working in under-resourced environments, where therapy strategies may need to be adapted to accommodate limited infrastructure, multilingual communities and alternative communication methods. Addressing these disparities in SLH training is essential to ensure that graduates are equipped to provide equitable, effective care across both urban and rural settings.

The Health Professions Council of South Africa (HPCSA) and other bodies have stressed the importance of providing culturally and linguistically relevant SLH services, but meaningful integration within SLH curricula and clinical settings has been slow to materialise (HPCSA, 2019; Khoza-Shangase & Mophosho, 2021). Additionally, students and practitioners often report discomfort and inadequacy in working with interpreters and language brokers – a common need in South Africa’s multilingual context (Khoza-Shangase & Kalenga, 2023; Mdlalo et al., 2016; Pascoe et al., 2018). This inadequacy raises concerns about the efficacy of SLH services and points to a gap in the curriculum where students could be trained more thoroughly in cross-cultural communication, interpretation practices and the use of linguistically appropriate diagnostic tools.

Beyond the curriculum, clinical service provision within SLH is equally affected by the colonial legacy that shaped South African healthcare services (Abrahams et al., 2019; Watermeyer & Neille, 2022). Traditionally, SLH services were predominantly available in urban, affluent areas, accessible only to a privileged few. This exclusivity is still evident in the accessibility of SLH services today, where the majority of South Africans, particularly those in rural or under-resourced areas, struggle to access care that respects their linguistic and cultural backgrounds. Undergraduate students training in SLH programmes are thus exposed to clinical experiences that may not fully reflect the diverse, multilingual and multicultural realities of South Africa (Abrahams et al., 2023). This disconnect can undermine their preparedness to practice in public health settings that serve the majority black population, as current clinical training often lacks comprehensive exposure to African languages, indigenous perspectives and community-based care approaches (Seabi et al., 2014).

The challenges to service provision in SLH are closely intertwined with the curriculum’s lack of focus on Afrocentric and culturally responsive approaches (Abrahams et al., 2019, 2023; Mdlalo et al., 2016; Mophosho et al., 2022). Evidence shows that SLH training programmes often do not provide students with resources tailored for the South African context, such as culturally relevant diagnostic and therapeutic tools (Khoza-Shangase & Kalenga, 2024a; Mophosho, 2018). A key limitation in addressing this gap is the lack of locally developed, validated materials, as most current resources are adapted from Western contexts, often without rigorous testing for cultural and linguistic appropriateness in South African populations. Given this challenge, there is a pressing need for targeted research and funding initiatives to support the development, adaptation and validation of contextually appropriate training materials. Without dedicated investment in research to create and evaluate these resources, SLH training programmes will continue to rely on materials that may not fully meet the needs of diverse South African communities. Lessons can be drawn from other contexts where collaborations between academic institutions, government agencies and professional bodies have been instrumental in funding research to generate locally relevant tools for health professions education (Hyter & Salas-Provance, 2021; Stockman et al., 2008). Establishing similar initiatives in South Africa will be crucial in ensuring that SLH education produces graduates equipped with the tools necessary for effective, equitable service delivery. Without training programmes addressing this challenge, students enter clinical placements unprepared to meet the needs of indigenous language speakers or to manage cross-cultural nuances effectively (Mophosho, 2018). This discrepancy is further complicated by power dynamics within the SLH profession, where most practitioners and educators are white, creating an environment that may inadvertently perpetuate cultural hegemony (Khoza-Shangase, 2019; Seabi et al., 2014). The resulting experience for black students can include feelings of exclusion and a sense that their own cultural backgrounds are undervalued within the profession (Abrahams et al., 2023).

The lack of transformation is not only in the curriculum content but also in the demographic composition of both students and academic and clinical training staff within SLH departments (Abrahams et al., 2023; Pillay et al., 2020). Although there has been a gradual increase in black students entering SLH programmes, representation among staff remains low. This demographic imbalance affects curriculum delivery and the mentorship environment, limiting the scope of cultural competence and diversity awareness that can be imparted to students. Scholars argue that the homogeneity within SLH staff and teaching materials reinforces a ‘single-story narrative’ that prioritises Eurocentric knowledge systems and methodologies, hindering the development of a curriculum that is genuinely reflective of South Africa’s diverse population (Khoza-Shangase & Mophosho, 2018).

Transformation within SLH curricula and clinical training changes this legacy by creating pathways that are inclusive of African perspectives, languages and community-centred healthcare approaches. This will require a decolonisation of both the content taught and the pedagogical methods employed in SLH training. As part of this transformation, SLH students advocate for an expansion of resources that go beyond translated materials from Western contexts (Hlayisi et al., 2024; Khamis & Bortz, 2023), suggesting a pressing need for locally developed assessment and intervention tools that align with South African cultural and linguistic contexts. Students have also voiced the importance of a more dynamic approach to cultural diversity in clinical placements, which should foster not only cultural awareness but also linguistic competency across the country’s eleven official languages (Khoza-Shangase & Kalenga, 2024b).

In summary, the demand for transformation in SLH education and clinical training is rooted in the need to dismantle historical inequities and create an SLH profession that aligns with the cultural and linguistic realities of the country. The experiences of SLH students highlight the need for a curriculum and clinical framework that equip future practitioners with the skills to deliver inclusive, contextually relevant and culturally competent care. Addressing these gaps will not only enhance the quality of SLH services but also empower students to participate fully in shaping a healthcare landscape that is reflective of and responsive to the diverse South African population, thus the value of this study. This study seeks to illuminate undergraduate SLH students’ experiences of transformation within both the curriculum and clinical service provision. By exploring their perceptions and experiences, this research aims to contribute to the ongoing discourse on how SLH education can be more responsive to the sociocultural and linguistic realities of South Africa, promoting a more equitable and inclusive professional landscape.

## Methodology

### Aim

The aim of this study was to investigate South African SLH undergraduate students’ experiences of transformation in the curriculum and clinical service provision.

### Objectives

The study addressed the following specific objectives:

- To explore undergraduate SLH students’ experience of transformation in the curriculum.
- To explore undergraduate SLH students’ experience of transformation in clinical service provision.

### Study design

This study utilised a cross-sectional, descriptive survey design to explore South African undergraduate SLH students’ experiences of transformation within their curriculum and clinical service provision (Babbie, 2020). Given the exploratory nature of the study, this design was chosen to capture a snapshot of (2020–2023 period) student perceptions and experiences across multiple institutions, allowing for a comprehensive view of the present state of SLH education transformation in South Africa. Both quantitative and qualitative data were collected using a web-based questionnaire, allowing for a rich, mixed-methods approach to data analysis.

### Study setting and participants

The study was conducted across multiple South African universities that offer accredited undergraduate programmes in SLH, as recognised by the HPCSA. Representation from all South African SLH training institutions was an important goal to ensure that findings reflected the diversity of SLH education in the country, considering institutional differences in curriculum design, student demographics and clinical training structures. Through a purposive sampling approach (Babbie, 2020), the study targeted undergraduate students in their third and fourth years of study, as they were expected to have had significant exposure to both academic coursework and clinical training, making them well-positioned to reflect on curriculum and service transformation in SLH education. First- and second-year students were excluded, as their limited exposure to clinical placements and the curriculum may have restricted their ability to fully assess experiences related to transformation. The study aimed to recruit a target of 60 participants across six institutions to obtain a broad range of perspectives. However, because of varying institutional participation and student response rates, the final sample consisted of 48 students from four universities. Recruitment was attempted at two additional institutions, but these did not yield participants because of various reasons such as logistical challenges, including administrative delays in research approval processes and difficulties in disseminating the survey link to students through official university channels. To enhance representativeness despite this limitation, recruitment efforts were intensified at the four participating institutions, ensuring inclusion of students from diverse racial, linguistic and socioeconomic backgrounds. Because of logistical constraints and privacy concerns at some institutions, precise demographic data on the overall SLH student population were not accessible.

### Sampling method

A non-probability purposive sampling method was employed to recruit participants who were deemed to have relevant experiences and insights on the study topic (Howell, 2002). Inclusion criteria focused on third- and fourth-year SLH students to capture those with adequate academic and clinical experience. Recruitment was facilitated through communication with department heads (HODs) or coordinators at each participating institution, who were asked to disseminate the online survey link to eligible students. While purposive sampling may limit generalisability, it was considered appropriate for an exploratory study examining a focused topic, and it ensured that participants were directly relevant to the research questions. This approach provided rich, context-specific insights that contribute to the existing literature on transformation in SLH education in South Africa.

### Data collection instrument

Data were collected through a 48-item online questionnaire designed specifically for this study, using Qualtrics, a secure online survey platform. The questionnaire consisted of both closed- and open-ended questions to allow for quantitative analysis of trends and qualitative exploration of personal experiences. The development of the questionnaire was informed by a review of existing literature on curriculum transformation, cultural competence in SLH education and higher education decolonisation efforts. Additionally, key themes from previous studies on SLH training in South Africa guided the formulation of items to ensure alignment with known challenges in the field. To ensure content validity, the questionnaire was reviewed by a panel of experts in SLH education, curriculum development and transformation research. Their feedback led to refinements in question wording, clarity and alignment with study objectives. Pilot testing was conducted with five postgraduate SLH students who had prior experience with the undergraduate curriculum, allowing for further adjustments to improve readability, relevance and response clarity. While formal psychometric validation was not conducted, efforts were made to ensure that the tool adequately captured students’ perceptions and experiences in a structured manner. Future research could strengthen the validity and reliability of this tool by conducting factor analysis and test-retest reliability assessments to further refine its use in similar studies on curriculum transformation.

The survey was structured into four main sections:

#### Demographics

Collected data on participants’ age, gender, race, language proficiency and year of study to contextualise responses and explore potential demographic influences on experiences of transformation.

#### Curriculum transformation

Explored students’ perceptions of transformation within the SLH curriculum, including the inclusion of Afrocentric content, local languages, cultural competence training and perspectives on whether the curriculum reflected South African demographics and values.

#### Clinical service provision transformation

Examined student experiences during clinical placements, including their exposure to diverse client populations, training in culturally and linguistically appropriate care and perceived preparedness to work with South African communities.

#### Overall perceptions and recommendations

Provided an open-ended section where students could express additional thoughts or recommendations regarding curriculum and clinical service transformation in SLH education.

### Data collection procedure

Prior to participant recruitment, formal requests for institutional permission were submitted to the relevant departments at each university. Approval was sought from either the Registrar’s office or the Heads of Speech-Language Therapy and Audiology departments, depending on institutional requirements. In all cases, permission was contingent on obtaining ethics clearance from the participating institutions before students could be contacted. Once approval was granted, department heads facilitated the recruitment process by distributing the study information and survey link to eligible students via institutional email lists and learning management systems. A standardised briefing was conducted virtually through telephonic discussions with department representatives at each institution to explain the study’s objectives, ethical considerations and the voluntary nature of participation. In all cases, information was also shared via email correspondence. Students who received the survey invitation were provided with an information sheet detailing the study, confidentiality measures and their right to withdraw at any stage without consequences. Participants accessed the survey through a unique, anonymous link, ensuring their responses were not personally identifiable. Two follow-up reminders were sent via institutional communication channels to maximise participation.

### Data analysis

#### Quantitative analysis

Using SPSS (Version 26), descriptive statistics, including frequency counts and percentages, were used to summarise participant demographics and perceptions of curriculum transformation. Additionally, demographic data were cross tabulated to explore potential patterns or associations by race, language proficiency and university (Howell, 2002). A Chi-square test was conducted to examine associations between students’ language proficiency and their perceived preparedness for diverse clinical practice. While Chi-square analysis provided insights into categorical relationships, the study did not conduct regression analysis because of the sample size limitations. Future research with a larger sample could employ logistic regression to assess the predictive strength of demographic factors (e.g. language proficiency, institutional affiliation) on students’ confidence levels and perceptions of transformation. This would allow for a deeper understanding of the extent to which specific factors influence preparedness for diverse clinical settings.

#### Qualitative analysis

Responses to open-ended questions were analysed using thematic analysis, following Braun and Clarke’s (2006) six-phase framework. After familiarisation with the data, initial codes were generated inductively. These codes were then grouped into patterns, and recurring concepts were clustered to form preliminary themes. Through an iterative process, themes were refined, reviewed and categorised based on conceptual similarities and relevance to the research objectives. Final themes were validated through researcher discussions and inter-rater agreement, ensuring that they accurately represented students’ experiences of curriculum and clinical service transformation, which were then captured in a report. The qualitative analysis was supported by NVivo software to assist in organising and coding data efficiently.

Three researchers independently coded the qualitative data to ensure reliability and to minimise individual bias in theme identification. Inter-rater reliability was assessed using Cohen’s kappa, which yielded a substantial agreement (κ = 0.78), indicating a strong level of consistency in coding decisions. Discrepancies were discussed and resolved through consensus to enhance the trustworthiness of the findings. Emerging themes focused on students’ perceptions of inclusivity in the curriculum, cultural representation in clinical placements and their overall sense of preparedness for practice in diverse South African communities. Representative quotes were extracted to illustrate key themes and highlight personal experiences.

### Rigour and trustworthiness

To ensure rigour and trustworthiness in this study, multiple strategies were employed to enhance credibility, dependability, transferability and confirmability (Babbie, 2020; Howell, 2002). Credibility was bolstered through triangulation of data sources, using both quantitative and qualitative data to provide a well-rounded perspective on SLH students’ experiences of transformation. The use of pilot testing with postgraduate SLH students further ensured clarity and relevance in the questionnaire items, strengthening content validity. Dependability was supported by a transparent and structured methodology, including a consistent approach to data collection and analysis, as well as detailed documentation of each stage in the research process. For transferability, the study’s context, sample characteristics and participant demographics were described comprehensively, enabling others to assess the relevance of findings to similar settings or populations. Confirmability was maintained through reflexivity; the researchers engaged in regular self-reflection to recognise and mitigate potential biases. Additionally, the thematic analysis of qualitative data was conducted independently by three researchers, with discrepancies resolved through consensus, ensuring that the themes were genuinely derived from participant responses rather than researcher assumptions. These combined efforts contribute to the overall trustworthiness and reliability of the study’s findings.

### Data management

Data management in this study was handled systematically to ensure the security, organisation and integrity of the collected information (Filkins et al., 2016). All data were collected anonymously through an online survey platform (Qualtrics), which securely stored responses on a password-protected server accessible only to the research team. Quantitative data from closed-ended questions were exported directly to SPSS software for statistical analysis, while qualitative responses to open-ended questions were imported into NVivo software for thematic analysis. Throughout the data analysis process, careful attention was paid to maintaining data accuracy and consistency. Backup copies of the dataset were stored on a secure, encrypted drive to prevent data loss. Following ethical guidelines, no identifiable information was collected, ensuring participant confidentiality. Once the analysis was complete, raw data were archived securely for potential future review, as per the institution’s data retention policy, and will be retained for 5 years in compliance with research data management regulations.

### Ethical considerations

This study adhered to the ethical guidelines stipulated by the University of the Witwatersrand’s Human Research Ethics Committee (Non-Medical) and was approved (number H19/09/01). Participation was entirely voluntary, with informed consent obtained from each student at the beginning of the online survey. The survey included an introduction outlining the purpose of the study, ensuring that participants understood their rights to anonymity, confidentiality and the option to withdraw without penalty. Data were collected anonymously, with no identifiable information linked to responses. All data were stored on a secure, password-protected server accessible only to the research team. Participants were informed that aggregated findings would be shared with their institutions for potential use in furthering curriculum and clinical service transformations.

## Results

### Demographic profile of the sample

The study sample consisted of 48 undergraduate students enrolled in third- and fourth-year SLH programmes across four South African universities. The majority of the sample (83.3%) was female, with male students comprising a smaller proportion (16.7%), reflecting the typical gender distribution observed within health sciences programmes in South Africa. In terms of racial demographics, black African students represented the largest group, making up 45.8% of the participants. This was followed by Indian students at 20.8%, coloured students at 16.7% and white students at 16.7%. Regarding the distribution of students by academic year, 45.8% were third-year students, while 54.2% were in their fourth and final year of study. Language proficiency data highlighted the linguistic diversity within the sample. English was the primary language of proficiency for 62.5% of the participants, while 37.5% reported proficiency in one or more indigenous South African languages in addition to English. Overall, the demographic profile of this sample reflects a diverse group of SLH students, representing different racial and linguistic backgrounds, which provides a nuanced perspective on their experiences with cultural and linguistic transformation in South African SLH education.

### Experience of transformation in the curriculum

This section presents the findings on students’ experiences of transformation within the SLH curriculum, specifically regarding culturally relevant content, Afrocentric perspectives and the inclusion of South African languages.

A significant number of students expressed dissatisfaction with the level of cultural and linguistic representation in the curriculum. Approximately 58% reported that the curriculum minimally reflected South African cultural and linguistic diversity, while 42% felt that it did not reflect diversity at all.

Table 1 provides a summary of students’ perceptions of curriculum relevance and transformation. The findings in Table 1 indicate that a significant proportion of students perceive the current SLH curriculum as lacking sufficient transformation in terms of cultural and linguistic relevance. A majority of participants felt that African languages and Afrocentric perspectives were not adequately integrated into their training, with many expressing concerns about the curriculum’s Eurocentric focus. Additionally, while some students acknowledged partial efforts towards cultural competence training, the overall consensus was that the curriculum does not fully prepare them for working in South Africa’s diverse linguistic and cultural landscape.

**TABLE 1:** Perceptions of transformation in the curriculum (*N* = 48).

Curriculum aspect	Percentage		
	Yes	No	Partially
Inclusion of African languages	18.8	66.7	14.6
Afrocentric perspectives in course content	20.8	62.5	16.7
Preparation for diverse linguistic contexts	29.2	54.2	16.6
Cultural competence training	31.3	47.9	20.8

These quantitative findings align with qualitative reports, where students expressed frustration over the Eurocentric focus of the curriculum. For example, one participant stated:

‘The curriculum doesn’t address the reality of our South African context. It feels like we’re studying in another country.’ (Participant 12, black African, third-year)

This indicates that numerical trends reflect deeper concerns about the curriculum’s lack of cultural relevance and inclusivity.

Two main themes emerged from the qualitative responses: (1) lack of representation in curriculum content and (2) insufficient cultural competence training.

#### Theme 1: Lack of representation in curriculum content

Many students felt that the curriculum did not adequately reflect South Africa’s cultural and linguistic diversity, but instead was predominantly focused on Western perspectives, neglecting Afrocentric approaches. This lack of representation often left students feeling disconnected from their own cultural backgrounds and unprepared to work within diverse South African communities. Students highlighted the absence of Afrocentric approaches, South African languages and contextually relevant materials, underscoring a perceived gap between their training and the realities they would encounter in practice:

‘The curriculum doesn’t address the reality of our South African context. It feels like we’re studying in another country.’ (Participant 12, black person, third-year)

‘There’s almost no emphasis on African languages or culturally relevant assessment tools, and it feels like my background is invisible here.’ (Participant 27, black person, fourth-year)

#### Theme 2: Insufficient cultural competence training

Participants reported that cultural competence training within their SLH programmes was minimal and largely theoretical, offering limited preparation for real-world interactions with culturally diverse clients. Students expressed a desire for more practical training, such as workshops and simulations, that could help them navigate cultural nuances and language barriers effectively. Many felt that current cultural competence training does not provide enough practical guidance on working with interpreters or understanding cultural subtleties:

‘We have some lectures on cultural competence, but they’re very theoretical and don’t prepare us for real-life interactions with patients who don’t speak English.’ (Participant 8, white person, fourth-year)

‘The curriculum needs more on working with interpreters and handling language differences in therapy. Right now, we’re not prepared for that.’ (Participant 3, Indian person, fourth-year)

### Experience of transformation in clinical service provision

This section explores students’ experiences with transformation in their clinical service provision, particularly their exposure to diverse client populations and the training they received for culturally and linguistically competent care.

Table 2 presents students’ self-reported levels of preparedness for working with linguistically and culturally diverse populations. The data reveal that a majority of students (60%) felt only ‘somewhat prepared’, indicating uncertainty about their ability to effectively engage with clients from diverse backgrounds. Notably, 30% of students reported feeling ‘unprepared’, suggesting significant gaps in their training related to cultural and linguistic competence. In contrast, a smaller proportion of students felt ‘very prepared’, with confidence levels appearing to be higher among those proficient in an indigenous language.

**TABLE 2:** Preparedness for diverse clinical service provision by language proficiency.

Preparedness level	Percentage	
	Indigenous language proficiency	English only
Very prepared	35.7	10.0
Somewhat prepared	57.1	60.0
Unprepared	7.1	30.0

Inferential statistics were used to examine the relationship between students’ language proficiency and their confidence in managing culturally diverse clinical encounters. A Chi-square test indicated a significant association between students’ indigenous language proficiency and their confidence levels in these settings (χ^2^\Chi^2χ^2^ (2, *N* = 48) = 7.84, *p* < 0.05), with students proficient in an indigenous language feeling more prepared than those who were not. To further interpret the strength of this relationship, Cramer’s V effect size was calculated, yielding V = 0.40, indicating a moderate effect. Additionally, 95% confidence intervals (CIs) were applied to preparedness levels to provide a more precise estimate of the proportion of students who felt unprepared or somewhat prepared. For students who felt ‘unprepared’, the 95% CI ranged from 18.5% to 41.5%, while for those who felt ‘somewhat prepared’, the CI ranged from 47.1% to 72.9%. These intervals suggest a moderate degree of variability in students’ self-reported preparedness, reinforcing the need for improved curriculum strategies to strengthen their confidence in clinical service delivery.

The quantitative findings suggest that language proficiency influences clinical confidence, a point echoed in qualitative responses. Several students who were not proficient in indigenous languages reported struggling to communicate with clients. One participant shared:

‘I often feel underprepared to handle therapy with clients who don’t speak English, which makes the clinical experience frustrating for both of us.’ (Participant 28, white person, third-year)

This reinforces the need for curriculum changes that incorporate indigenous language training to improve student preparedness.

Four themes emerged from the qualitative responses: (1) resource scarcity in clinical settings, (2) language barriers in clinical practice, (3) call for practical cultural competence training and (4) desire for indigenous knowledge and Afrocentric approaches.

#### Theme 1: Resource scarcity in clinical settings

Students highlighted a significant shortage of culturally and linguistically appropriate materials during their clinical placements. They often found themselves relying on English-language resources, which limited their ability to provide effective care for non-English-speaking clients. This lack of resources created challenges in making accurate assessments and providing fair interventions, leading to feelings of inadequacy and frustration:

‘It’s hard to find resources or assessment tools for African language speakers. We often have to use tools that aren’t relevant, and it doesn’t feel fair to the clients.’ (Participant 19, Indian person, third-year)

‘We need more resources that reflect the South African population, not just English speakers.’ (Participant 11, black person, fourth-year)

#### Theme 2: Language barriers in clinical practice

Language barriers were a recurring issue for many students, who felt unprepared to interact effectively with clients who spoke indigenous languages. The absence of training in local languages or guidance on working with interpreters left many students feeling under-resourced for multilingual clinical interactions. Students pointed out the need for language training and more practical instruction on how to navigate linguistic differences within therapy:

‘Without adequate training in local languages, it’s challenging to communicate effectively with clients. We’re not taught how to handle situations where an interpreter is needed.’ (Participant 5, white person, fourth-year)

‘I often feel underprepared to handle therapy with clients who don’t speak English, which makes the clinical experience frustrating for both of us.’ (Participant 28, white person, third-year)

#### Theme 3: Call for practical cultural competence training

Students reported that they lacked hands-on training to navigate culturally diverse clinical scenarios. Students expressed a strong need for hands-on cultural competence training that could prepare them for real-life clinical scenarios, to help them work more confidently with clients from different linguistic backgrounds. They recommended incorporating role-plays, community placements and practical simulations that reflect diverse cultural and linguistic contexts within South Africa. Such training, they argued, would better equip them for navigating cultural and linguistic diversity in clinical practice:

‘We need more opportunities to work with interpreters and adapt our techniques for non-English speakers.’ (Participant 10, Indian person, third-year)

‘The curriculum should include more real-life scenarios that teach us to handle language differences and navigate cultural contexts effectively.’ (Participant 17, white person, fourth-year)

#### Theme 4: Desire for indigenous knowledge and Afrocentric approaches

Many participants advocated for the inclusion of indigenous knowledge and Afrocentric approaches in their curriculum, feeling that such perspectives would make their training more relevant to the South African context. Students suggested adding content on African languages, cultural practices and local health beliefs, which could enhance their cultural competence and respect for clients’ backgrounds:

‘We need more content on African languages, even basic training in one or two to help with understanding our clients.’ (Participant 33, Indian person, fourth-year)

‘Including African languages in the curriculum would make our training more relevant and respectful of our clients’ backgrounds.’ (Participant 21, black person, fourth-year)

## Discussion

This study provides valuable insights into South African SLH undergraduate students’ experiences of transformation within their curriculum and clinical service provision. The findings highlight several critical areas for improvement in SLH education, notably in terms of incorporating culturally relevant content, Afrocentric perspectives and linguistic diversity. These results reveal both gaps and opportunities within SLH programmes to better prepare students for the realities of practicing in a diverse, multilingual society, aligning with South African higher education’s overarching transformation goals.

The demographic profile of the sample reflects the diversity of students enrolled in South African SLH programmes. The predominance of female participants (83.3%) aligns with global trends in SLH education, where the profession remains female-dominated (Lindsay & Kolne, 2023). The racial distribution of participants – with black African students representing the largest group (45.8%), followed by Indian students (20.8%), coloured students (16.7%) and white students (16.7%) – suggests increasing diversity within SLH training programmes, which is essential for addressing the transformation agenda in higher education (Khoza-Shangase & Mophosho, 2018). The linguistic profile of participants indicates that 62.5% were primarily English-speaking, while 37.5% reported proficiency in one or more indigenous South African languages. This linguistic distribution is particularly relevant, given the study’s focus on curriculum transformation and cultural competence, as it suggests that a significant portion of students may not receive formal training in indigenous languages, despite the multilingual nature of SLH service delivery in South Africa. Lastly, the inclusion of both third-year (45.8%) and fourth-year (54.2%) students ensures that perspectives from students with substantial academic and clinical experience are captured, providing insight into how transformation efforts are perceived at different stages of training. Overall, while the sample is diverse, the underrepresentation of certain linguistic groups highlights ongoing challenges in achieving fully inclusive SLH training.

### Experience of transformation in the curriculum

The majority of students reported that the curriculum fell short in reflecting South African cultural and linguistic diversity. Specifically, over 60% of participants felt that African languages and Afrocentric perspectives were largely absent from their training. The lack of representation in curriculum content was highlighted by students who felt that Afrocentric perspectives and South African languages were insufficiently incorporated. As students shared, this gap in the curriculum created a sense of ‘otherness’ and reinforced a disconnection between their training and the communities they would serve. The qualitative responses indicate a strong student desire for a more inclusive curriculum, reflective of local languages, contexts and values. This disconnect is compounded by the historical exclusion of black South Africans from SLH professions, where Western epistemologies have long dominated the field (Moonsamy et al., 2017). These findings align with previous research identifying a Eurocentric bias in SLH programmes in South Africa (Kathard & Pillay, 2013; Khoza-Shangase & Mophosho, 2018) – highlighting similar Eurocentric biases in SLH curricula. It is for such findings that Kathard and Pillay (2013) call for an ‘Africanisation’ of SLH, where educational content not only accommodates indigenous knowledge but also challenges existing colonial structures in education. These findings highlight the pressing need for a curriculum review to incorporate Afrocentric content and multilingual training, ensuring that future SLH professionals are equipped to serve the country’s diverse population.

The exclusion of culturally and linguistically diverse content in SLH curricula is not unique to South Africa. In the United States, Stockman et al. (2008) identified similar gaps, where SLH programmes historically lacked representation of African American Vernacular English and bilingual language development. Likewise, Hyter and Salas-Provance (2021) note that global SLH education has primarily been structured around Western linguistic norms, limiting students’ ability to provide equitable services to culturally diverse populations. This suggests that South African SLH education can benefit from international models that actively integrate culturally responsive content, including community-based learning and multilingual training approaches.

The theme of insufficient cultural competence training in the curriculum also aligns with broader calls for the integration of culturally responsive care in SLH education (Moonsamy et al., 2017). While SLH students reported some exposure to cultural competence, the training was often theoretical and limited in practical application. Only 31.3% of students felt their training sufficiently covered cultural competence. This aligns with Mophosho (2018) and Ullauri (2021), who highlighted the importance of integrating cultural and linguistic competence into SLH curricula to improve patient-centred care, particularly in multilingual settings. The call for practical and contextual training is especially critical, given South Africa’s complex linguistic landscape, where 11 official languages and numerous cultural groups coalesce. Students in this study emphasised the need for modules that address how to work effectively with interpreters and navigate language differences, both essential skills for future SLH practitioners. Mdlalo et al. (2016) and Khoza-Shangase and Kalenga (2023) have previously highlighted the challenges of working with interpreters in SLH settings in South Africa.

Limited practical training in cultural competence is a global challenge in SLH education. Kamhi (2011) argues that cultural competence training in the United States has often been treated as an elective rather than a core competency, resulting in graduates who feel underprepared to engage with linguistically and culturally diverse clients. Similarly, Hyter and Salas-Provance (2021) emphasise the need for experiential learning – such as immersion programmes and case-based simulations – to bridge the gap between theoretical instruction and real-world application. These insights reinforce the need for South African SLH programmes to adopt more practical, hands-on approaches to cultural competence training, including the use of simulated patient interactions and role-playing exercises.

### Experience of transformation in clinical service provision

Student experiences in clinical service provision reflected significant gaps in their ability to work with linguistically and culturally diverse clients. Quantitative findings indicated that a considerable portion of students (30%) felt unprepared to work with non-English-speaking clients, a result confirmed by the statistically significant relationship between indigenous language proficiency and perceived preparedness. Students proficient in an indigenous language reported feeling more confident in clinical settings, correlating with Chi-square test findings of higher confidence levels in clinical interactions, suggesting that language proficiency is a critical factor in student readiness. The finding of resource scarcity, particularly in assessment tools and intervention materials for non-English speakers, reflects challenges previously documented by Khoza-Shangase and Mophosho (2018) and Mdlalo et al. (2016), who highlighted the limited availability of culturally relevant SLH tools for indigenous language speakers. Students in this study echoed these concerns, sharing that they often relied on resources that were linguistically and culturally misaligned with their clients’ needs. This lack of resources raises significant ethical questions regarding the accuracy and fairness of SLH assessments and interventions in South Africa’s multicultural context. It also raises opportunities for investing in technological advances as far as the use of machine learning, artificial intelligence and large language models is concerned (Madahana et al., 2022; Tu et al., 2024).

The qualitative theme of language barriers highlights the need for SLH training that better equips students to work in multilingual clinical settings. Students reported feeling unprepared and, at times, frustrated by language challenges, an experience that has been similarly documented in studies on healthcare provision in multilingual contexts (Mdlalo et al., 2016). Given the multilingual landscape of South Africa, where only 9.6% of the population cites English as their first language (Wigdorowitz et al., 2022), training SLH students in basic proficiency in local languages or in strategies for effectively working with interpreters is crucial. Without these skills, students may feel underprepared, and clients may experience barriers to effective communication and care.

Multilingualism presents a challenge in SLH training worldwide. In Australia and Canada, where diverse linguistic populations exist, clinical training programmes have incorporated interpreter training and bilingual service models to improve accessibility (Horton & Munoz, 2021; Hsieh, 2016; Ng et al., 2022). Additionally, Caesar (2004) notes that SLH clinicians in the United States often rely on ad hoc interpreters because of a lack of formalised multilingual training. These global examples highlight the need for South African SLH curricula to move beyond theoretical discussions of multilingualism and actively train students in managing interpreter-mediated sessions, cross-cultural communication and language-specific assessment tools.

The identified need for enhanced cultural competence training is consistent with findings from Abrahams et al. (2019), who argued that the SLH profession in South Africa continues to employ a predominantly Western model that inadequately addresses the cultural realities of African clients. As seen in this study, students called for practical training that moves beyond theoretical discussions of cultural sensitivity and engages with real-life clinical scenarios. Such training could involve simulations (Hewat et al., 2020; Nagdee et al., 2022), role-plays and community placements in diverse areas, which have been shown to improve cultural competence and confidence in clinical practice (Hyter & Salas-Provance, 2021).

Participants emphasised the importance of integrating indigenous knowledge, Afrocentric approaches and language diversity within SLH training, which would align with the government’s post-apartheid education transformation mandates as outlined in the 1997 Education White Paper on Transformation of Higher Education. This would also support the Department of Health’s Batho Pele principles, advocating for patient-centred, contextually relevant healthcare services. The theme of indigenous knowledge integration within the curriculum aligns with Pentecost et al. (2018), who argue that the decolonisation of SLH must encompass not only content but also the underlying values and pedagogies, which is similar to Hyter and Salas-Provance’s (2021) advocacy for decolonisation of SLH education and integration of Afrocentric models. Students in this study suggested introducing language courses and modules that focus on African health perspectives, a recommendation echoed by Kathard and Pillay (2013). This approach would not only respect the cultural backgrounds of clients but would also serve to ‘re-humanise’ SLH education by valuing African perspectives, rather than presenting Western frameworks as superior.

Students’ call for Afrocentric approaches reflects the larger movement towards decolonisation in South African higher education, exemplified by student movements such as #RhodesMustFall and #FeesMustFall. Afrocentric approaches to SLH practice, as students suggested, could be achieved through partnerships with traditional healers and community leaders, promoting a health model that is contextually appropriate and respected by local communities (Hyter & Salas-Provance, 2021; Pentecost et al., 2018). Afrocentric models could enrich the SLH field by providing culturally resonant solutions to communication and hearing disorders, thus fostering greater trust and collaboration with clients. Lastly, the need for training in linguistic diversity was a key theme, as students expressed frustration over limited opportunities to develop proficiency in indigenous languages or to gain skills in working with interpreters. This finding is consistent with Mdlalo et al. (2016) and Mophosho (2018), who reported that SLH services in South Africa are often compromised by language mismatches between therapists and clients. Integrating language training within SLH curricula would prepare students to serve clients more effectively and respectfully, enhancing the quality and accessibility of care for non-English-speaking South Africans. The call for decolonising SLH education aligns with international discussions on diversifying health professions curricula. While South Africa faces unique post-apartheid challenges, the movement towards indigenising education has also been explored in Canada and Australia, where universities have integrated indigenous perspectives into medical and allied health training (Horton & Munoz, 2021). Pillay and Kathard (2015) draw parallels between South African and global decolonisation efforts, emphasising the need for SLH education to incorporate not only linguistic diversity but also indigenous healing practices and culturally relevant service delivery models. This underscores that transformation in SLH education must be both contextually relevant and informed by successful international decolonisation models.

This study has several limitations that should be acknowledged. The use of purposive sampling and a relatively small sample size may limit the generalisability of findings. The sample consisted of 48 participants from four universities, which, while diverse, may not fully represent the broader population of SLH students across all South African training institutions. The exclusion of students from two targeted institutions further limits national representativity. Additionally, differences in curricula, resource availability and training models across institutions may have influenced student experiences, potentially impacting the findings. Future studies should account for these variations by comparing institutional policies and training approaches to provide a more nuanced understanding of curriculum transformation. To improve generalisability, future research should aim for a larger, more representative sample by including all SLH training institutions in South Africa. Multi-institutional collaborations could facilitate broader participation and ensure the inclusion of students from historically disadvantaged institutions, rural-based universities and institutions that did not participate in this study, which may provide fuller experiences of curriculum and clinical training transformation. Additionally, longitudinal studies tracking students’ perspectives over time would provide a more comprehensive understanding of how transformation efforts evolve throughout their training and into professional practice, improving on the current cross-sectional approach design that captured a single point in time. Expanding the methodological approach to include focus groups and in-depth interviews could further enrich the findings by capturing nuanced student experiences that may not be fully expressed through survey responses alone.

Lastly, as the study relied on self-reported data, responses may be subject to social desirability bias or personal interpretation of transformation concepts. Participants may have responded in a way they perceived as more acceptable rather than fully reflecting their experiences, which could have influenced the data. Future research could mitigate this by incorporating anonymous interviews or mixed-method approaches to balance self-report with observational or secondary data sources.

## Conclusion

This study highlights the significant gaps and opportunities for transformation within South African SLH undergraduate education, particularly in curriculum content and clinical service provision. Findings reveal that while students in SLH programmes are enthusiastic about serving South Africa’s diverse populations, they often feel underprepared because of a curriculum that remains heavily Western-oriented and lacks sufficient focus on South African languages, Afrocentric perspectives and culturally relevant clinical tools. The scarcity of linguistically and culturally appropriate resources in clinical settings, combined with limited training in cultural competence and working with interpreters, leaves many students feeling unprepared for real-world practice, particularly in under-resourced, multilingual communities. While the findings of this study highlight the specific challenges faced by South African SLH students, similar struggles have been documented in global SLH education. The need for curriculum transformation, greater linguistic inclusivity and improved cultural competence training is evident in multiple international contexts, including the United States, Canada and Australia. These global perspectives provide valuable insights that can inform the transformation of SLH education in South Africa, emphasising the importance of adopting best practices from international curricula while ensuring that local cultural and linguistic realities remain central to training.

To bridge the identified gaps, this study calls for a comprehensive transformation in SLH education that aligns with South Africa’s diversity and the government’s broader objectives for higher education reform. Integrating Afrocentric approaches and indigenous knowledge into SLH curricula is essential to fostering a more inclusive and culturally responsive field. Afrocentric models and content can serve to ‘re-humanise’ the curriculum, affirming the cultural backgrounds of clients and dismantling the dominance of Western frameworks that do not fully address the health and communication needs of African communities. To this end, five key practical recommendations are being advanced in this article. Firstly, SLH programmes must develop and implement Afrocentric course modules. Further research is needed to inform *curriculum revision and Afrocentric integration* in SLH education. While there is growing recognition of the need for culturally relevant training, rigorous studies should be conducted to explore effective ways of incorporating Afrocentric theories and perspectives into SLH curricula. This research should assess the feasibility, effectiveness and impact of adapting Western frameworks to the South African context, particularly in expanding the curriculum to reflect the country’s cultural and linguistic diversity. Evidence-driven approaches should guide the development of modules on local health beliefs and practices, which could involve collaborations with traditional healers, community leaders and academic experts in these areas. Establishing such partnerships, informed by empirical research, will ensure that curriculum transformation efforts are both culturally meaningful and pedagogically sound. This can be operationalised through the integration of case studies, clinical scenarios and community-based projects that reflect South African cultural contexts.

Secondly, indigenous language training must be formally integrated into the curriculum, where *incorporation of indigenous language training* occurs. Basic proficiency in indigenous languages, or at minimum exposure to linguistic variations within South African languages, should be incorporated into SLH curricula. Language training should also include interpreter skills and guidelines on working with language facilitators to support non-English-speaking clients. This could be implemented through short courses, language immersion programmes or interprofessional training with linguistic experts to ensure students acquire practical communication skills for multilingual settings. Thirdly, cultural competence training should move beyond theory to include practical, contextualised experiences, where *enhanced cultural competence and practical training* become standard practice. Cultural competence training should move beyond theoretical instruction to involve practical simulations, community placements and role-play scenarios reflecting diverse cultural and linguistic interactions. Such training will prepare students for real-life encounters in clinical settings and help them navigate cultural nuances effectively. One possible implementation strategy is the use of simulated patient encounters where students practice culturally responsive care in a controlled environment before entering clinical settings. Fourthly, *development of locally relevant resources* should be expanded through multidisciplinary collaborations. There is an urgent need to create assessment tools and intervention materials that are culturally and linguistically appropriate for South African clients. This can be achieved through research grants and funding initiatives that support the development and validation of new tools specific to South African contexts. Collaborative projects among SLH departments, linguistic, engineering, arts and technology departments, professional bodies and local communities could drive the development of resources that reflect South Africa’s linguistic diversity. Fifthly, *stakeholder collaboration and policy development* must be continuously strengthened to sustain transformation. Educational institutions, the HPCSA and professional bodies should work together to implement these changes and ensure that the SLH curriculum and clinical training and continued professional development align with South Africa’s unique cultural and linguistic landscape. Practical mechanisms to support this include forming a national SLH curriculum review task force and establishing annual policy review forums involving students, educators and community representatives to assess the effectiveness of ongoing changes. Regular feedback from students, graduates and community stakeholders should guide curriculum updates and ensure that educational practices remain responsive to the evolving needs of South African society.

In summary, transforming SLH education in South Africa is essential to creating a workforce equipped to provide equitable, culturally competent and linguistically relevant services. Addressing these educational and clinical gaps will empower future SLH practitioners to serve South Africa’s diverse populations more effectively and uphold the principles of inclusivity and social justice central to the nation’s healthcare mission. Implementing these changes will require a strategic, multi-stakeholder approach that combines research, policy development and practical training initiatives to drive sustainable transformation.
